# Time-Domain Impedance Analysis on Passivation Quality of 316L Stainless Steel with Portable-Probe-Measured Potential Step Transient

**DOI:** 10.3390/ma18143276

**Published:** 2025-07-11

**Authors:** Haobin Li, Bufan Jiang, Chi Cheng, Congqian Cheng, Qibo Wang, Tieshan Cao, Jie Zhao

**Affiliations:** School of Materials Science and Engineering, Dalian University of Technology, Dalian 116024, China; lhbshuai6@mail.dlut.edu.cn (H.L.); 196890141@mail.dlut.edu.cn (B.J.); cc13592291263@mail.dlut.edu.cn (C.C.); wangqi_0504@yeah.net (Q.W.); tieshan@dlut.edu.cn (T.C.); jiezhao@dlut.edu.cn (J.Z.)

**Keywords:** 316L stainless steel, potential step method, time-domain impedance, quality of passivation, three-electrode

## Abstract

To achieve rapid detection of stainless steel passivation quality, a time-domain impedance method was investigated based on a potential step transient with a portable three-electrode probe. A comparison of the effects of signal analysis and transient parameters was conducted, and the results were compared with those obtained in a bulk solution with a general three-electrode system. The measured transient current with the probe offered a higher signal-to-noise ratio, with minimal deviation from the frequency-domain impedance observed at a step amplitude of 0–100 mV. Measurements using the three-electrode probe under different stabilization times indicated that, after 30 s of stabilization, the measurement deviation was less than 1%, enabling a rapid assessment. Comparative testing of surfaces with varying passivation quality revealed that the pitting potential increases with increasing time-domain impedance, demonstrating the method’s capability to distinguish passivated surfaces with different corrosion resistances.

## 1. Introduction

Austenitic 316L stainless steel is widely used in the aerospace, urban rail transit, nuclear power, and pharmaceutical industries due to its good corrosion resistance and mechanical properties [1,2,3]. The good corrosion resistance of such steel depends on the formation of a dense and protective passive film on its surface. To satisfy the requirements of corrosion resistance in severe environments, many pretreatment methods including chemical passivation and new electrochemical treatment have been proposed for promoting the quality of protective passive films [4,5,6]. However, the on-site passivation quality usually differs from the reported experimental work due to complicated component shapes and different operation environments, resulting in corrosion issues [7,8]. Thereafter, measurements of passivation quality during the manufacturing and on-site service management is critically necessary for stainless steel components, especially for those used in severe corrosive environments.

The primary methods for evaluating the passivation quality of stainless steel are those recommended by the Chinese National Standards and the ASTM standard, such as the blue dot test [9]. However, these approaches suffer from limitations, including poor reagent stability and potential damage on the surface [10]. The International Electrotechnical Commission (IEC) has previously recommended electrochemical impedance spectroscopy as an alternative [11,12]. This method offers high accuracy and imparts nondestructive characteristics to the surface, making it a promising candidate for passivation film assessments. Nonetheless, practical engineering experience has shown that electrochemical potential-based detection methods often require long testing durations and exhibit significant signal fluctuations [13]. Therefore, it remains a significant challenge for achieving rapid and reliable evaluations of surface passivation quality.

The potential step time-domain impedance method can measure the dynamic impedance of an electrochemical system by applying a unit step potential, enabling rapid and nondestructive testing [14,15,16,17,18]. This technique has garnered significant research interest in applications such as rapid impedance measurements and health assessments of lithium batteries, electrochemical immunoassays, and corrosion impedance spectroscopy measurements of carbon steel [19,20,21,22]. There are few reports on whether the potential step impedance is applicable to the passivation quality inspection of stainless steel. However, this method was only tested in bulk solution environments in various fields, which does not demonstrate its ability to be applied to engineering field sites. Field surveys indicate that three-electrode probes are commonly used for coating detection at engineering sites [23,24]. However, the bulk solution detection system is quite different from the three-electrode probe regarding electrode structure and testing environment. It is still unknown whether the potential step time-domain impedance method that is suitable for bulk solution systems can remain stable and reliable within the three-electrode probe environment.

To facilitate quick in-situ assessments of passivation quality for stainless steel in manufacturing settings, this research team has systematically validated the feasibility of using the potential step time-domain impedance method to measure passivation film impedance within a three-electrode system [25]. Furthermore, a dedicated three-electrode probe was designed to support portability and field adaptability. Using nitric acid-passivated 316L stainless steel, this study investigated whether the aforementioned method is applicable in a three-electrode probe environment. The discussion focuses on the potential step amplitude, which has the greatest impact on the method. Additionally, the effects of different stabilization times and surface qualities on the measurement results are examined to assess the practical engineering application potential of the method.

## 2. Materials and Methods

### 2.1. Testing Method

**Figure 1:**
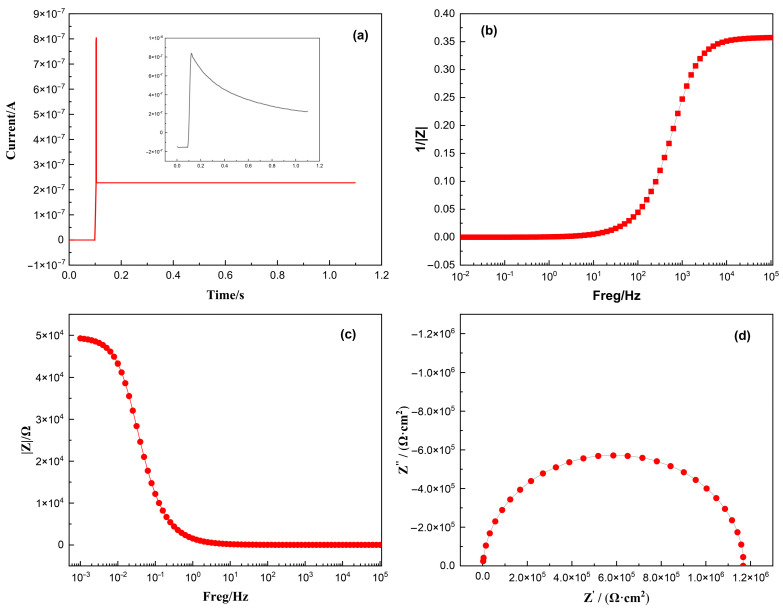
Schematic diagram of data processing: (**a**) current diagram of timing ampere response in simulation system; (**b**) admittance diagram obtained after Fourier transform of data in (**a**); (**c**) Bode plot; and (**d**) Nyquist plot.

**Figure 2:**
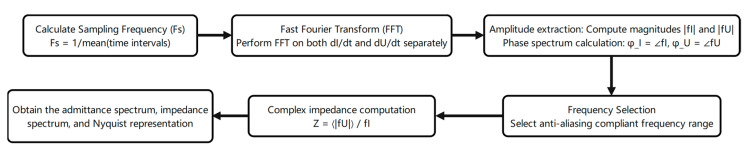
Data processing workflow.

According to the principle of electrochemical time-domain impedance, the impedance of any time-invariant system can be determined by applying an excitation signal and collecting the corresponding response signal. Through a series of mathematical transformations, the system’s impedance can be calculated [26]. Under the potential step condition, if the potential step signal replaces the unit pulse signal, the amplitude of all harmonic components will be equal to 1 after Fourier transformation analysis. Therefore, impedance can be directly obtained using Equation (1). Therefore, the impedance value can be calculated through simply collecting the response current signal of the system according to Equation (1). The measured response signal (Figure 1a) was Fourier-transformed to yield the admittance–frequency plot (Figure 1b). Admittance inversion produced the impedance–frequency relationship (Bode plot, Figure 1c), while plotting the real vs. imaginary impedance components generated the Nyquist plot (Figure 1d), enabling a determination of the system impedance. [27,28] The data processing workflow is illustrated in Figure 2.

(1) $$\Omega \left(j\omega \right)=\frac{F[E\left(t\right)]}{F[I\left(t\right)]}=\frac{1}{I(j\omega )}$$

where *E*(*t*) is the differentiated voltage, *I*(*t*) is the differentiated current, *F* is the Fourier transform, and *I*(*jω*) is the current after differentiation and Fourier transform.

Assuming that surface dispersion effects are negligible, the admittance plot is converted into a Nyquist plot using an RC equivalent circuit. This measurement method exhibits heightened susceptibility to noise interference due to its inherently small signal magnitude. To mitigate this, the current response signal undergoes noise reduction through several techniques, including adjacent averaging, the Savitzky–Golay method [29,30], and FFT filtering [31]. Following that, a variable window-width smoothing method is applied, where the window size increases exponentially, with the index ranging from 1 to N/5 (where N is the total number of data points), ensuring effective signal smoothing.

### 2.2. Experimental Materials and Methods

A 2 mm thick 316L stainless steel sheet was first cut into 20 × 20 mm^2^ samples using wire electrical discharge machining, which was performed using the AP200 wire electrical discharge machine (EDM), manufactured by Sodick Co., Ltd., Japan. The samples were polished with sandpaper and cleaned with alcohol, and then passivated in HNO_3_ solution according to ASTM A380 standards [32]. To achieve different levels of passivation quality, the samples were passivated for 5–45 min at varying nitric acid concentrations and passivation temperatures.

**Figure 3:**
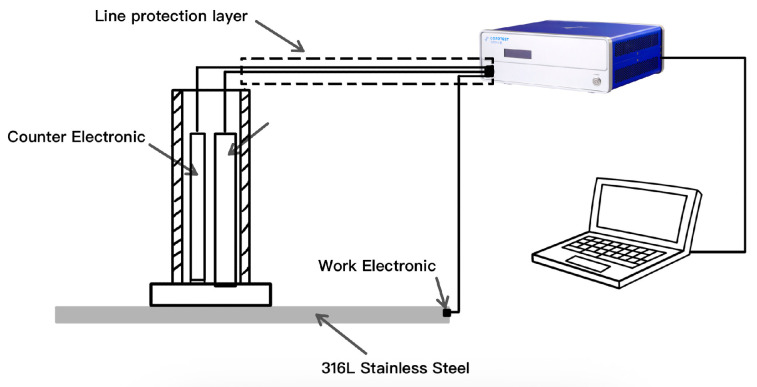
Schematic diagram of three-electrode probe testing system.

Impedance and polarization tests using a three-electrode system were conducted on a CS350 electrochemical workstation, manufactured by Wuhan CorrTest Instruments Corp., Ltd., located in Wuhan, China. This experiment employed a flat-panel electrolytic cell and a dedicated three-electrode probe as show in Figure 3, with a working area of 1.0 cm^2^.

The reference electrode and counter electrode were Ag/AgCl and Pt, respectively. A sodium citrate–citric acid buffer solution with pH = 6.5 was used as the testing solution in both the potential step and conventional frequency electrochemical impedance tests to maintain a stable pH and ionic conductivity during both the potential step and electrochemical impedance spectroscopy (EIS) measurements. The Ag/AgCl reference electrode provided a stable reference potential, while the inert Pt counter electrode ensured current conduction without introducing side reactions. The potential step test utilized a potential step amplitude range from 0 to 150 mV relative to the open-circuit potential, with a sampling frequency of 10 kHz and a maximum sampling duration of 1 s. In the conventional frequency-domain impedance measurements, the sinusoidal disturbance potential amplitude was 10 mV, and the frequency range was from 10 kHz to 0.01 Hz. To analyze corrosion resistance under different passivation states, potentiostatic polarization tests were performed with a scan rate of 0.5 mV/s in a 3.5% NaCl solution. Each test condition was repeated with 3–5 samples. The sample surface was then imaged using SEM, which was performed using the JSM-IT800 scanning electron microscope, manufactured by JEOL Ltd., Japan. And ImageJ software (ImageJ 1.54g) was used to measure the damaged surface percentage.

## 3. Results and Discussion

**Figure 4:**
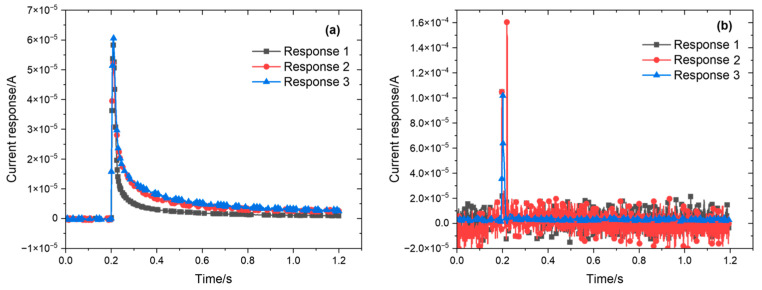
Comparison of current response measurements (**a**) using a three-electrode probe and (**b**) in bulk solution.

Two tested environments were considered to compare the differences in current response measurements of the 316L stainless steel that was passivated for 30 min at 35 °C in 8% HNO_3_ solution: the bulk solution and the three-electrode probe setup. The potential step amplitude was set to 0–100 mV, with a measurement time of 1 s, a sampling frequency of 10 kHz, and a system stabilization time of 10 min for each step. As shown in Figure 4a, under potential step polarization conditions, the current initially increases sharply to a peak before rapidly decreasing in the bulk solution environment. This current response curve is also accompanied by significant noise. On the other hand, little or no noise was seen in the current response signal obtained with the three-electrode probe, and the signal-to-noise ratio was significantly higher than that of the flat-panel electrolytic cell (Figure 4b).

**Figure 5:**
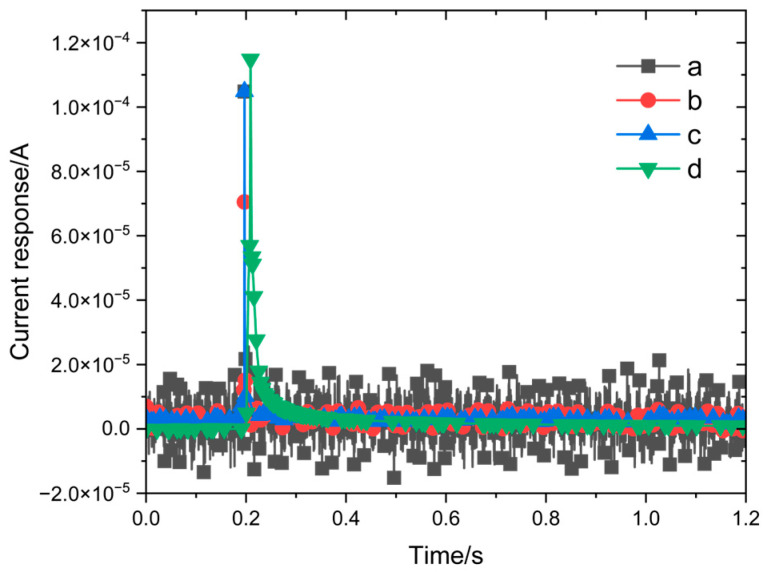
Smoothing and denoising of response signals: (a) raw data of bulk solution; (b) denoising by adjacent averaging method for bulk solution; (c) denoising by variable-window smoothing filter method for bulk solution; and (d) raw data of three-electrode system.

The response signal must undergo smoothing and denoising before subsequent data processing. The effects of different smoothing and denoising methods are displayed in Figure 5. It can be observed that the data from the bulk solution environment are difficult to fully smooth, and all processing methods affect the peak height of the current response to varying degrees. By contrast, the response signals obtained in the three-electrode probe environment are already close to the smoothed results, requiring a simpler denoising process and exhibiting less impact on peak characteristics.

**Figure 6:**
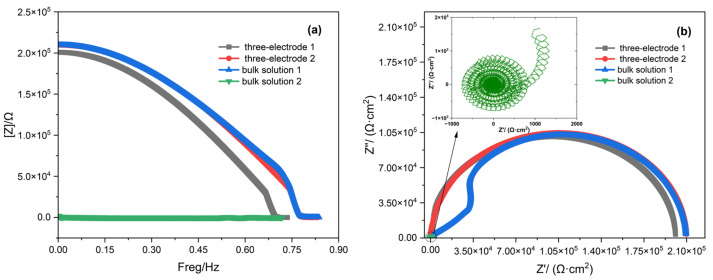
Data processing plots: (**a**) Bode plot and (**b**) Nyquist plot.

The processed data can be converted into a Bode plot through differentiation, Fourier transform, and reciprocal calculation. The mathematical transformation of the Bode plot data yields impedance spectra (as shown in Figure 6). All test data were obtained from measurements of different samples subjected to the same passivation method under the aforementioned experimental conditions. However, the accuracy of the impedance spectra is affected by the height of the response peaks, leading to significant deviations in bulk solution environments. Additionally, in such environments, the lower signal-to-noise ratio (SNR) often results in unusable measurements.

As shown in Figure 6, both three-electrode 2 and bulk solution 1 provide accurate impedance measurement results. However, for bulk solution 1, the sample preparation and sealing processes increase surface roughness on unmeasured areas, causing localized current inhomogeneity at the working electrode edge periphery. This induces fluctuations in both the Bode and Nyquist plots, though it does not compromise the overall impedance measurement validity. Given the inherent instability of electrochemical impedance measurements and their susceptibility to multiple influencing factors, certain measurement deviations, such as those observed in three-electrode 1 caused by variations in working electrode surface roughness and solution concentration discrepancies, remain within acceptable tolerance limits. Regarding bulk solution 2, excessive environmental noise prevents the step response signal from being smoothed, rendering the resulting Bode plot and Nyquist plot entirely unusable. By contrast, the three-electrode probe system demonstrates a significantly higher SNR. This improvement can be attributed to the following factors: The three-electrode probe incorporates an anti-static interference cable, which prevents electrostatic interference from affecting the measurement results. The three-electrode probe takes measurements while it is in close contact with the stainless steel being tested, resulting in a more stable overall structure. Additionally, the solution volume involved in the three-electrode probe is small, making it easier to form a stable double layer and effectively reduce fluctuations in charge transfer impedance [33,34]. By contrast, bulk solution measurements are subject to more influencing factors and exhibit greater instability. These factors significantly enhance the stability and signal-to-noise ratio of the three-electrode probe measurements.

**Figure 7:**
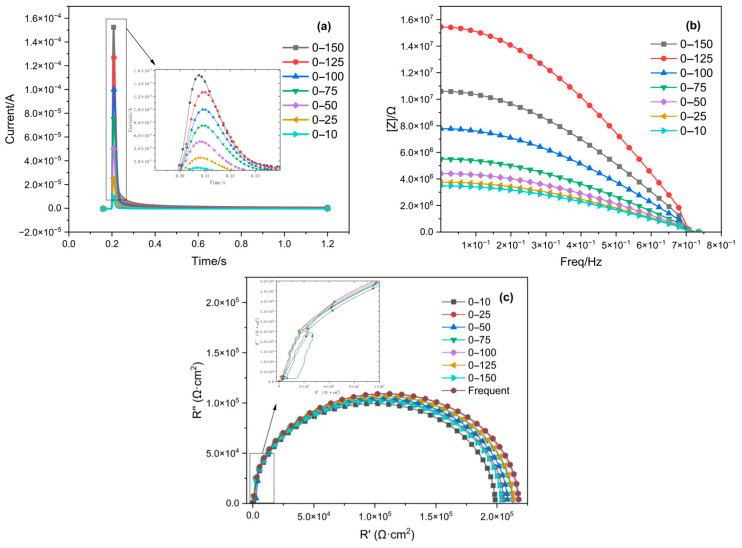
Impedance spectra obtained by fitting step potential responses of different orders, along with the denoised response current signals and impedance spectra obtained from different step potentials (10–150 mV): (**a**) response current signal; (**b**) Bode plot; and (**c**) Nyquist plot.

A comparison of the impedance spectra fitted from the response current signals of the 316L stainless steel, which was passivated for 30 min in an 8% HNO_3_ solution at 35 °C under different step potential conditions and testing environments, is shown in Figure 7. For the results measured with the three-electrode probe, the Bode plot and impedance spectrum gradually converge with the frequency-domain impedance spectrum as the step amplitude increases at step potentials below 100 mV. However, when the step potentials are greater than 100 mV, the deviation between the Bode plot and the impedance spectrum from the frequency-domain impedance spectrum increases as the step amplitude rises. Combining the measurement results from both the three-electrode probe and the bulk solution, the impedance spectrum fitted from the measurements with a step amplitude of 0–100 mV is closest to the frequency-domain impedance spectrum. Table 1 lists the impedance values fitted at different step amplitudes and their errors compared to the frequency-domain impedance values.

**Figure 8:**
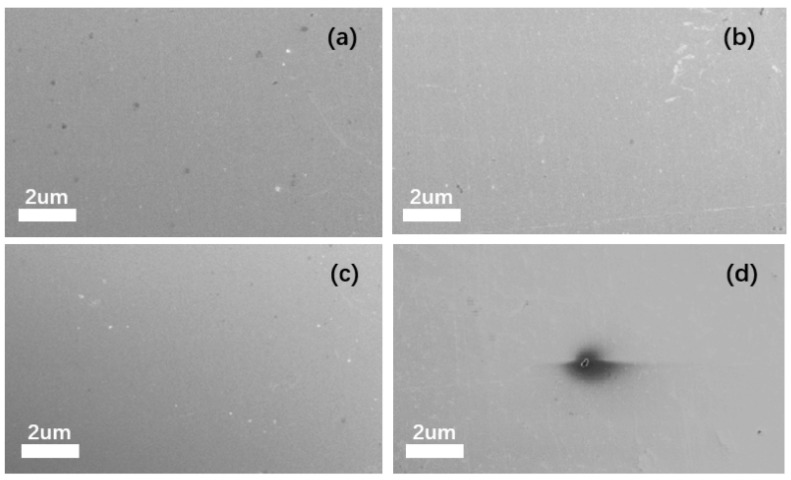
SEM images of samples with step amplitudes: (**a**) SEM image of 0–50 mV amplitude step response; (**b**) SEM image of 0–75 mV amplitude step response; (**c**) SEM image of 0–100 mV amplitude step response; and (**d**) SEM image of 0–125 mV amplitude step response.

After observing all the step amplitude samples via SEM, it was found that when the step amplitude does not exceed 100 mV, no pitting occurs on the sample surface, as in the steps less than 100 mV (shown in Figure 8a,b,c). However, when the step amplitude exceeds 100 mV, pitting corrosion is observed on the sample surface, as in the 0–110 mV step (shown in Figure 8d). The image displays a representative sample surface. The pitting area of all samples exhibiting pitting corrosion was measured, revealing a pitting area percentage of 1.38 ± 0.73% under this measurement method.

**Figure 9:**
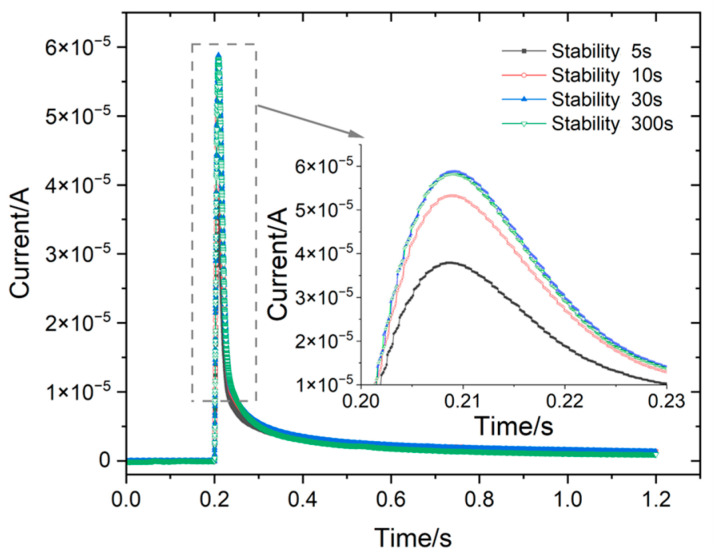
Current response measured by three-pole probes at different stabilization times.

Figure 9 shows the current response measured by the three-electrode probe at different stabilization times. Notably, the peak current response exhibits significant differences at different stabilization times. The shorter the stabilization time, the smaller the peak value of the current response peak. Nevertheless, no discernible deviation in the peak value was observed when the stabilization time reached 30 s or more.

**Figure 10:**
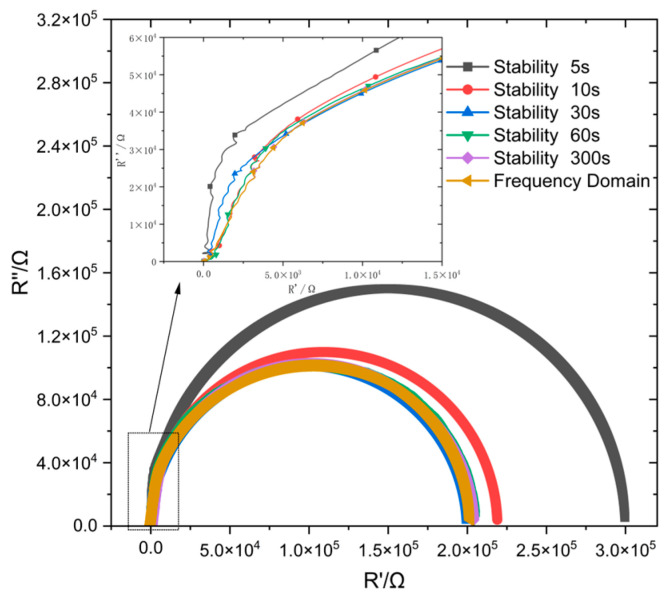
Comparison of potential step time-domain impedance and frequency-domain impedance at different stabilization times.

Figure 10 displays the fitted impedance spectra derived from potential steps at different stabilization times. At a stabilization time of 5 s, the solution fails to form a stable double electron layer on the surface of the passivation film, resulting in a significant deviation between the measured impedance spectrum and the frequency-domain impedance spectrum.

By contrast, when the stabilization time exceeds 30 s, a high similarity between its impedance spectrum and the frequency-domain impedance spectrum was found, and the deviation value was less than 1% (Table 2). Moreover, further prolonging of the stabilization time gradually decreases this deviation value. This is much shorter than the time required for stabilization in a bulk solution environment, further enhancing the feasibility of this method for on-site engineering applications.

**Figure 11:**
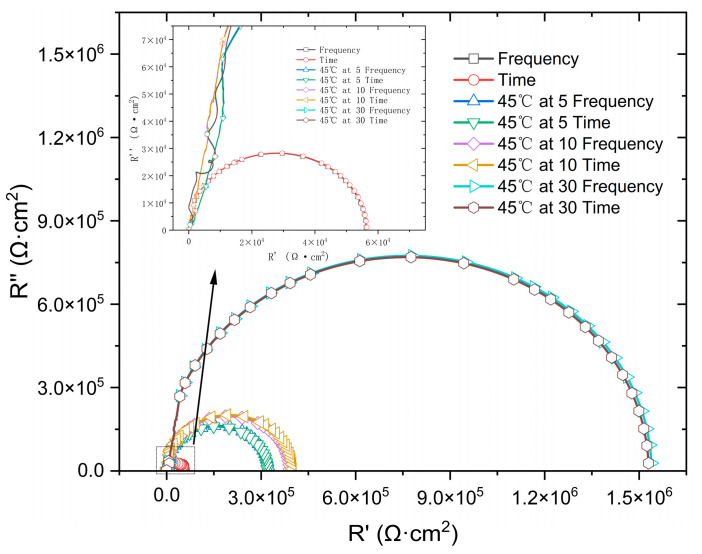
Comparison of potential step time-domain impedance and frequency-domain impedance under different passivation states.

**Figure 12:**
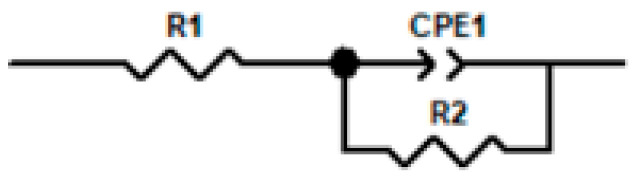
RQ equivalent circuit model.

Figure 11 presents a comparison between the time-domain impedance based on the potential step measured by a three-electrode probe at different surface qualities and the frequency-domain impedance measured by a flat electrolytic cell. The results were fitted with an RQ equivalent circuit (as show in Figure 12) to obtain parameters such as charge transfer impedance and solution resistance, as shown in Table 3. At 45 °C, the impedance value of the 316L stainless steel passivation film gradually increases with a prolonged immersion time, accompanied by excellent agreement between the time-domain impedance and frequency-domain impedance spectra.

**Figure 13:**
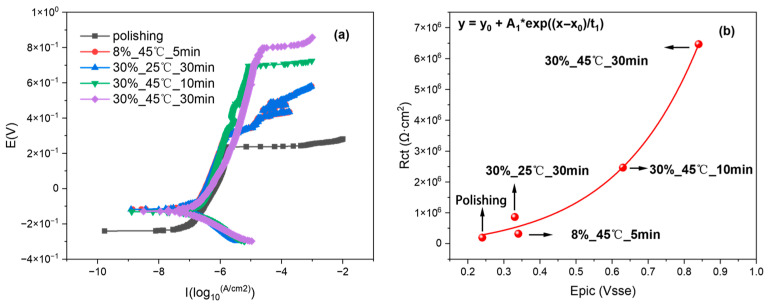
(**a**) Pitting potential curves of 316L stainless steel under different passivation treatments. (**b**) The relationship between pitting potential and impedance values of 316L stainless steel under different passivation treatments.

Potentiodynamic polarization tests were conducted on 316L stainless steel with different passivation states in a 3.5% NaCl solution. The impedance values were correlated with the pitting potentials, as shown in Figure 13. The impedance value and pitting potential show a near-linear correlation: increased pitting potential reflects higher 316L stainless steel impedance and enhanced corrosion resistance. As this falls outside the study’s scope, the mathematical specifics of this relationship are not elaborated. These results also demonstrate that the three-electrode probe system used for electrochemical impedance measurements is capable of accurately distinguishing between 316L stainless steel samples with varying passivation qualities. However, in the group passivated at 45 °C for 30 min, values of α > 1 were observed, which are physically meaningless in the context of the CPE model. This anomaly arises because the response signal generated by the time-domain impedance method is significantly smaller than the inherent noise of the electronic instrumentation, leading to an insufficient signal-to-noise ratio (SNR). Since CPE parameters are particularly sensitive to low-frequency data—where signal amplitudes are inherently weak and more susceptible to noise interference—any limitations in equipment resolution or improper grounding can cause phase drift to be misinterpreted as α > 1 during fitting.

## 4. Conclusions

This study introduced a new evaluation method for passivation quality using time-domain impedance analysis based on potential step transients. The usage of a portable probe and a traditional three-electrode system in bulk solution was compared, and the effect of various test parameters was investigated.

1. Compared to the traditional three-electrode system in bulk solution, the three-electrode probe system features a more integrated design, a more stable testing architecture, and fewer influencing factors. It requires a smaller volume of electrolyte, reducing external interference with the measurement results, thereby significantly improving the signal-to-noise ratio of the current response. When the potential step amplitude was maintained within a range of 0–100 mV, the deviation between time-domain and frequency-domain impedance measurements was minimized, ensuring greater measurement accuracy. Pitting corrosion will initiate when the potential step amplitude surpasses 100 mV.
2. The influence of stabilization time on the impedance value in the three-electrode probe test system was studied. Notably, a high similarity was observed between the time-domain impedance spectrum fitted from the measured response and the frequency-domain impedance spectrum when the stabilization time exceeded 30 s. This demonstrates that the time-domain impedance spectra can accurately measure the charge transfer impedance of passivated film, and the variation range of charge transfer impedance is small. With the extension of the stabilization time, the stability of the three-electrode probe system was further improved, and the measured time-domain impedance was closer to the frequency-domain impedance.
3. The influence of different surface qualities on the impedance value in the three-electrode probe test system was investigated. Under optimized test conditions of potential step (0–100 mV), measurement time (1 s), frequency (10 kHz), and stabilization time (≥30 s), the three-electrode probe system can effectively measure the impedance value and distinguish the different surface passivation qualities. Simultaneously, a clear positive correlation was observed between charge-transfer resistance and pitting potential, confirming that higher resistance values indicate improved corrosion resistance.

## Figures and Tables

**Table 1:** Impedance values fitted with step amplitudes ranging from 10 to 150 mV and their errors compared to frequency-domain impedance values.

Three-electrode Probe Measurement			Bulk Solution		
Test Methods	Impedance (Ω·cm^2^)	Deviation	Test Methods	Impedance (Ω cm^2^)	Error
Frequency-domain impedance	2.19 × 10^5^	/	Frequency-domain impedance	2.01 × 10^5^	/
10 mV	1.99 × 10^5^	9.1%	10 mV	1.00 × 10^5^	50.1%
25 mV	2.05 × 10^5^	6.1%	20 mV	1.44 × 10^5^	28.3%
50 mV	2.08 × 10^5^	4.8%	30 mV	1.59 × 10^5^	21.0%
75 mV	2.13 × 10^5^	2.8%	40 mV	1.71 × 10^5^	15.1%
100 mV	2.17 × 10^5^	0.8%	50 mV	1.94 × 10^5^	3.6%
125 mV	2.13 × 10^5^	2.5%	100 mV	2.04 × 10^5^	1.2%
150 mV	2.04 × 10^5^	6.6%	150 mV	2.06 × 10^5^	2.2%

**Table 2:** Deviation values of potential step time-domain impedance and frequency-domain impedance at different stable times.

Test Methods	Impedance (Ω·cm^2^)	Deviation
Frequency-domain impedance	2.02 × 10^5^	/
Stability 5 s	3.00 × 10^5^	48.2%
Stability 10 s	2.19 × 10^5^	8.3%
Stability 30 s	2.00 × 10^5^	0.9%
Stability 60 s	2.04 × 10^5^	1.0%
Stability 300 s	2.04 × 10^5^	0.7%

**Table 3:** The time-domain impedance values obtained by fitting the frequency-domain impedance under different passivation states with RQ equivalent circuit. Here, R_1_ represents the solution resistance, Q and α are CPE parameters simulating a double-layer capacitor, and R_2_ denotes the charge transfer impedance.

Sample Treatment Method	Measurement Method	*R*_1_ (Ω·cm^2^)	Q (μF cm^−2^s^α−1^)	α	*R*_2_ (100 K Ω·cm^2^)
Not passed	Frequency-domain impedance method	10.63	118.6	0.92	0.5
	Potential step method	10.80	120.4	1.0	0.5
45 °C nitric acid passivation 5 min	Frequency-domain impedance method	11.82	76.3	0.86	3.2
	Potential step method	11.49	79.6	0.95	3.2
45 °C nitric acid passivation 10 min	Frequency-domain impedance method	10.84	72.4	0.79	3.9
	Potential step method	10.59	79.5	0.82	3.9
45 °C nitric acid passivation 30 min	Frequency-domain impedance method	9.35	27.3	1.04	14.2
	Potential step method	9.47	25.7	1.06	14.3

## Data Availability

The original contributions presented in this study are included in the article. Further inquiries can be directed to the corresponding author.
